# Structured Cap-assisted Margin Assessment Reduces Residual Neoplasia after Colorectal Polypectomy: A Prospective Multicentre Proof-of-Concept Study

**DOI:** 10.1055/a-2900-1290

**Published:** 2026-07-03

**Authors:** Alexander Huelsen, Timothy Willis, Prabha Ketheson, Caroline Cooper, Jennifer Borowsky, Sooraj Pillay, Ian Paul Hughes, Zaki Hamarneh

**Affiliations:** 1 Department of Gastroenterology and Digestive Health 60093 Gold Coast University HospitalSouthportQueenslandAustralia; 2 Anatomical Pathology, Pathology Queensland 60093 Gold Coast University HospitalSouthportQueenslandAustralia; 3 Department of Gastroenterology and Hepatology 1966 Princess Alexandra HospitalBrisbaneQueenslandAustralia; 4 Faculty of Health, Medicine and Behavioural Science 1974 The University of QueenslandBrisbaneQueenslandAustralia; 5 Anatomical Pathology, Pathology Queensland 1966 Princess Alexandra HospitalBrisbaneQueenslandAustralia; 6 Research and Biostatistics Office 60093 Gold Coast University HospitalSouthportQueenslandAustralia

**Keywords:** endoscopy lower GI tract, polyps/adenomas/..., tissue diagnosis, endoscopic resection (polypectomy, ESD, EMRc, ...), diagnosis and imaging (inc chromoendoscopy, NBI, iSCAN, FICE, CLE...)

## Abstract

**Background and study aims** Recurrence after endoscopic resection of medium-to-large non-pedunculated colorectal polyps remains common and varies substantially between operators. We evaluated a structured cap-assisted resection margin assessment (CARMA) protocol designed to standardise near-field inspection and margin clearance.

**Patients and methods** Prospective multicentre study of 48 non-pedunculated colorectal polyps ≥10 mm (mean 22.2 mm) in 43 patients. Following conventional cold or hot snare resection, CARMA comprised structured cap-assisted near-field inspection with targeted cold-snare resection of margin abnormalities. Circumferential cold-snare resection (CS360) was performed for histologic validation. The primary outcome was immediate histology-confirmed residual neoplasia after standard resection and after CARMA. Secondary outcomes included recurrence at surveillance, adverse events and exploratory assessment of inter-operator variability.

**Results** After conventional resection, CARMA identified histology-confirmed residual neoplasia in 24/48 polyps (50.0%, 95% CI 35.9–64.1%). Following CARMA-targeted resection, residual microscopic neoplasia persisted in 3/48 (6.3%, 95% CI 2.1–16.8%; McNemar *p* = 9.5 × 10 ^−7^ ). According to ESGE criteria (adenoma or sessile serrated lesion with dysplasia ≥20 mm), 24/46 non-cancer lesions would have met recommendations for margin ablation; among the remaining lesions, CARMA identified histology-confirmed residual in 12/22 (54.5%). At surveillance (median 6 months), no recurrence was observed (0/38; 95% CI 0–9.3%). In an exploratory inter-operator analysis, residual rates differed before CARMA (20.0% vs 57.9%) and appeared to converge after CARMA (0% vs 7.9%). No CARMA- or CS360-related adverse events occurred.

**Conclusions** Structured cap-assisted margin inspection substantially reduced histology-confirmed residual neoplasia in this proof-of-concept study. These findings support evaluation of margin-focused inspection and clearance strategies in larger studies.

## Introduction

Colorectal cancer (CRC) remains a leading cause of cancer-related mortality, and complete removal of precursor lesions is central to its prevention. 1 2 3 4 A persistent contributor is marked inter-operator variability in incomplete resection and recurrence outcomes, exceeding three-fold even among experienced endoscopists, as demonstrated in the CARE study and large multicentre endoscopic mucosal resection (EMR) cohorts. 5 6 7 8 Despite advances in polypectomy and EMR techniques, with recurrence rates below 5% reported by some expert operators, recurrence after removal of medium to large non-pedunculated colorectal polyps (LNPCPs) persists in routine clinical practice at 15–30%. 7 9 10 11 12 13 These recurrences are predominantly driven by incomplete margin excision. 5 6 The burden may be underestimated, as prior studies have commonly relied on limited margin biopsies to assess incomplete resection – an approach that samples only a limited proportion of the resection margin. 5 7

Adjunctive thermal margin ablation techniques, including snare-tip soft coagulation and argon plasma coagulation, have therefore been introduced, with substantial reductions in recurrence to <5% demonstrated in large multicentre prospective studies, reinforcing the central role of margin residual disease. 6 14 However, outcomes remain operator-dependent, with real-world recurrence rates still reported at 6–16%. 15 16 17 18 Furthermore, current guidelines recommend thermal margin treatment for selected lesions and do not provide a routinely applied, visual verification endpoint across the full spectrum of lesions and resection modalities. 19 20

We hypothesised that some residual disease following standard resection may be endoscopically appreciable and resectable using structured, stabilised, near-field inspection. We therefore developed the cap-assisted resection margin assessment (CARMA) protocol, which incorporates targeted cold-snare resection of any abnormal or non-verifiable pit pattern identified under cap-assisted near-field view. In this study, a subsequent 2–3 mm circumferential Cold Snare 360° resection (CS360) was used to assess for residual neoplasia after the CARMA endpoint had been achieved.

The aim was to assess whether structured, non-thermal margin management strategies could improve margin clearance after colorectal polypectomy.

## Patients and Methods

### Study Design and Setting

This prospective multicentre study was conducted at two Australian tertiary referral centres between 2021 and 2024, evaluating the CARMA protocol and CS360 following conventional colorectal polyp resection. All procedures were performed by two experienced interventional endoscopists. The study was approved by the institutional Human Research Ethics Committees (HREC/2021/QMS/75076), prospectively registered (NCT05099432), and conducted in accordance with the Declaration of Helsinki. Written informed consent was obtained from all participants.

### Participants

Adults (≥18 years) undergoing colonoscopic resection of polyps ≥10 mm (medium-to-large non-pedunculated colorectal polyps; mean size 22.2 mm, range 10–45 mm) were prospectively enrolled. Case allocation reflected routine clinical scheduling. Up to two polyps per patient were eligible for inclusion. Exclusion criteria comprised pedunculated polyps, endoscopically suspected invasive cancer (referred for surgical management), recurrent scar lesions, inflammatory pseudopolyps, active inflammatory bowel disease, colostomy, and polyposis syndromes.

### Patient Preparation

All participants received split-dose bowel preparation. Antiplatelet and anticoagulant medications were managed in accordance with current guideline recommendations, with aspirin continued. 21 All procedures were performed under propofol sedation administered by an anaesthetist.

### Index Resection

Polyp size was estimated using snare diameter comparison. All polyps were assessed using high-definition white-light endoscopy (HDWL) and narrow band imaging (NBI), classified according to the Paris and JNET classification systems, and lesion location was documented. 22 23 24 The initial resection approach (cold or hot snare; en bloc or piecemeal) was selected by the endoscopist based on polyp morphology and location, in accordance with guideline recommendations, with the aim of achieving a macroscopically complete resection with a 1–2 mm margin of normal mucosa. 19 20

Submucosal lifting was performed using succinylated gelatine (Gelofusine; B. Braun, Crissier, Switzerland) mixed with dilute adrenaline (1:100,000–1:500,000) and methylene blue, unless omitted for small lesions. Dedicated cold snares (10 mm, Captivator Cold, Boston Scientific, Marlborough, USA or 9 mm, Exacto, Steris, Mentor, USA) were used including for all CARMA-directed resections and CS360. Hot snares (10–20 mm, Olympus, Tokyo, Japan or Boston Scientific, Marlborough, USA) were used when thermal resection was undertaken and for snare-tip soft coagulation haemostasis when required. Adjuvant thermal ablation of the post-resection margin was intentionally omitted as part of the study protocol.

All procedures were performed with high-definition or 4K colonoscopes (Olympus HQ190 or 1500 series colonoscopes, Tokyo, Japan or Fujifilm 700 series, Tokyo, Japan), fitted with a distal transparent cap (D-201-14304/D-201-12704; Olympus, Tokyo, Japan) under carbon dioxide insufflation.

### CARMA Imaging Requirements

CARMA requires stable near-field inspection for high-precision margin assessment. Near-field visualisation was achieved using endoscopes with short focal-length or close-focus optics, including Olympus Dual Focus “near-focus” mode and close-focus magnification on Fujifilm systems. Across all platforms, a distal cap protrusion of 2–2.5 mm beyond the camera plane provided mechanical stabilisation and a fixed near-viewing distance, enabling reproducible, high-resolution margin assessment while maintaining an adequate field of view.

### CARMA Protocol

**Fig. 1:**
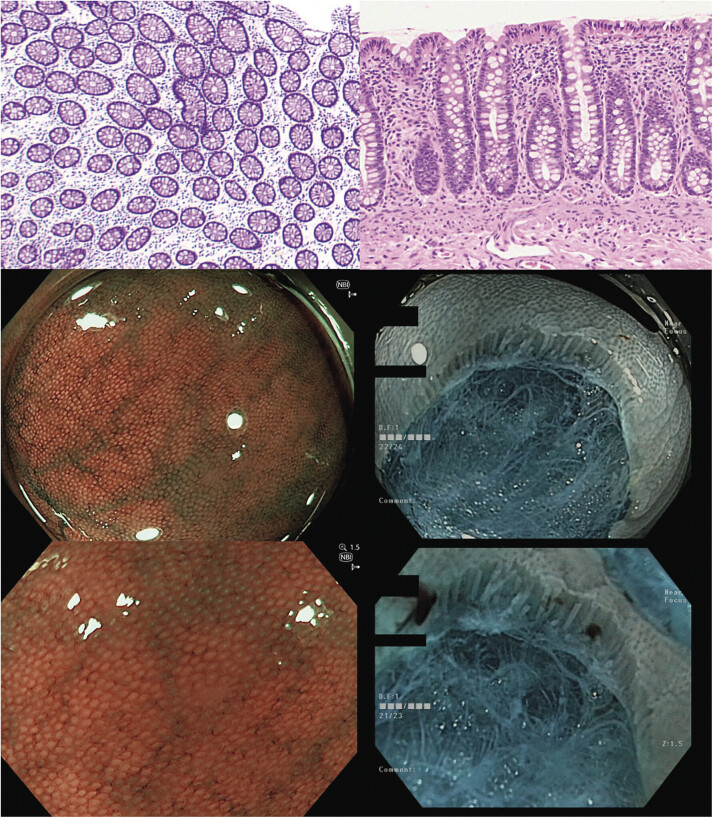
Normal colonic pit pattern (CARMA endpoint) and correlation with histologic crypt architecture. ( **A** ) En-face haematoxylin and eosin (H&E) section of normal colonic mucosa demonstrating circular crypt openings (pit pattern). ( **B** ) Longitudinal H&E section showing aligned tubular crypt architecture. ( **C** ) Endoscopic near-field en-face view demonstrating a normal circular pit pattern. ( **D** ) Endoscopic near-field longitudinal view confirming linear crypt alignment and a normal pit pattern. ( **E** ) Optional 1.5× optical magnification illustrating normal en-face pit architecture. ( **F** ) Optional 1.5× magnification verifying longitudinal crypt architecture at the margin. These images define the expected appearance of a circumferentially normal pit pattern margin, which represents the visual endpoint of CARMA-directed margin assessment and targeted cold-snare resection.

After the endoscopist deemed the index resection complete using standard inspection (high-definition or 4K white-light endoscopy ± NBI), CARMA was then applied in two predefined steps:

1. **Structured margin inspection:** The distal cap was gently apposed to the mucosal surface and rotated circumferentially to obtain a stable near-field view of the entire resection margin. Insufflation, irrigation, or water immersion of the cap was used as required to optimise pit-pattern assessment. Any suspicious base irregularities were evaluated in the same manner.
2. **Targeted cold-snare** ( **TCS** ) **resection:** Any margin area not demonstrating a normal colonic pit pattern, or suspicious for residual adenoma or sessile serrated lesion (SSL), was resected using a cold snare, including a 1–2 mm margin of normal colonic mucosa. Findings were classified as high-confidence (unequivocally abnormal) or low-confidence (margin areas in which a normal pit-pattern endpoint could not be confidently confirmed). The endpoint of CARMA was a circumferentially normal pit-pattern margin. Cap-assisted near-field inspection and pit-pattern assessment have been described in prior optical studies. 25 26 A representative correlation between normal histologic crypt architecture and the normal cap assisted near-field pit-pattern appearance defining the CARMA endpoint is shown in Fig. 1 .

### CS360 Protocol

Following completion of CARMA, a 2–3 mm circumferential cold-snare resection of the entire resection margin was performed. CS360 served both as histologic validation for residual microscopic neoplasia and allowed exploratory assessment of CS360 as a potential non-thermal margin clearance approach.

A single submucosal tattoo was placed approximately 3 cm distal to the resection site when location was judged likely to be difficult to identify at surveillance colonoscopy.

### Specimen Handling and Histology

The index lesion, CARMA-targeted specimens, and CS360 samples were retrieved in separate containers, with meticulous irrigation or exchange of tubing, suction traps, and snares between steps to minimise cross-contamination. CARMA-targeted and CS360 specimens underwent cut-to-extinction serial sectioning. All samples were reviewed by experienced gastrointestinal pathologists, with secondary review performed when clarification or confirmation was required. Procedural specimen category was available to pathologists, but CARMA confidence level and endoscopic impression were not communicated.

### Outcomes

#### Primary Outcome

The primary outcome was the immediate stepwise reduction in histology-confirmed residual neoplasia across two sequential stages:

1. Pre-CARMA: residual neoplasia following conventional polyp resection, identified by CARMA-directed targeted cold-snare resection.
2. Post-CARMA: residual neoplasia detected by circumferential CS360 resection, serving as histologic validation.

#### Secondary Outcomes

- Residual neoplasia rates in CARMA-negative margins, as assessed by CS360.
- Diagnostic accuracy of high-confidence versus low-confidence CARMA targets.
- Endoscopic and histologic recurrence at first surveillance colonoscopy, with biopsy or resection of any suspicious area.
- Descriptive pre- and post-CARMA residual neoplasia rates by lesion size category, resection technique and histology.
- Exploratory assessment of inter-operator variability in margin clearance pre- and post-CARMA.
- Procedure-related adverse events, including intraprocedural bleeding, delayed bleeding, post-procedural pain requiring clinical assessment and perforation.

### Follow-up

Participants underwent structured follow-up comprising electronic medical record review at 30 days and clinic assessment (in person or by telephone) as required. Surveillance colonoscopy was performed 4–12 months after resection in accordance with national guidelines. 27 At surveillance, the resection site was assessed using high-definition or 4K white-light endoscopy, NBI and near-field inspection. Targeted biopsies or cold-snare resection was performed when endoscopic features were suspicious. This selective approach reflects evidence demonstrating high concordance between meticulous optical scar assessment and histology, while avoiding the limitations of random biopsies, which sample only a small proportion of the resection defect. 22 28 29 30

### Statistical Analysis

Paired proportions of residual neoplasia before and after CARMA were compared using McNemar’s exact test. Absolute risk reduction with 95% confidence intervals (CIs) was calculated using the Wilson method. Subgroup analyses were descriptive. Because the study was not powered for subgroup comparisons and resection technique was selected clinically, pre- and post-CARMA residual neoplasia rates were summarised within clinically relevant subgroups to assess directional consistency rather than comparative efficacy. Positive predictive values (PPVs) and negative predictive values (NPVs) for CARMA confidence categories and recurrence rates were calculated with exact (Clopper–Pearson) 95% CIs. All statistical analyses were performed using SPSS Statistics (Version 26, IBM Corp., Armonk, NY, USA).

### Sample Size Justification

As a proof-of-concept study, we aimed to enrol approximately 50 polyps to detect a large, paired reduction in residual neoplasia. Published incomplete resection rates based on limited margin biopsies likely underestimate residual burden; we anticipated a higher prevalence approaching 40% when the resection margin was comprehensively sampled. 7 We anticipated that CARMA would reduce residual neoplasia to <10%. A paired reduction of >30% would require approximately 40 polyps for 80% power, or 53 polyps for 90% power, at a two-sided *α* of 0.05. Given the exploratory nature of this study and limited prior data for formal powering, these calculations were used to contextualise sample size rather than to define a confirmatory endpoint.

## Results

### Patient and Polyp Characteristics

**Fig. 2:**
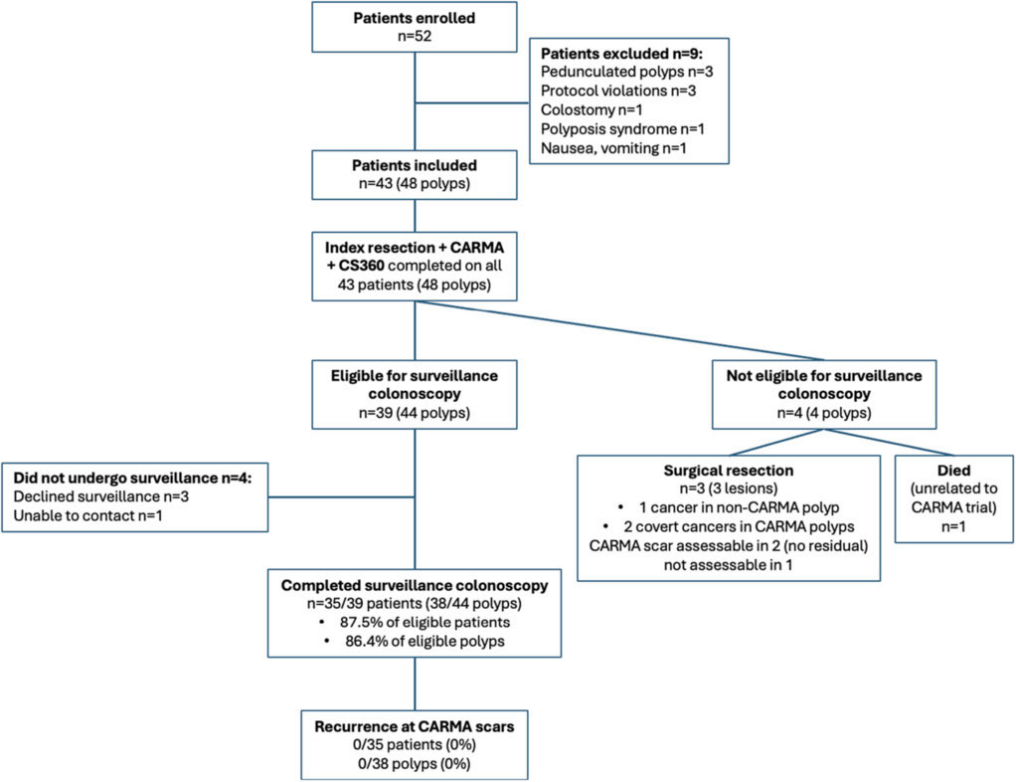
Study flow diagram.

Fifty-two patients were enrolled; after 9 exclusions, 43 patients with 48 non-pedunculated colorectal polyps ≥10 mm (mean 22.2 mm, range 10–45 mm) were analysed ( Fig. 2 ). The median patient age was 66 years (range 38–82). Histology comprised 37 adenomas (77.1%), 9 serrated polyps (18.8%) and 2 adenomas with a focus of covert cancer. Baseline patient and polyp characteristics are summarised in Table 1 .

**Table 1:** Baseline patient, lesion, resection and histologic characteristics (48 polyps in 43 patients).

Patient characteristics	
Patients, *n*	43
Median age (range), years	66 (38–82)
Sex, *n* (female/ male)	20/23
**Lesion Characteristics**	
Polyps, *n*	48
Polyp size, median (range), mm	20 (10–45); mean 22.2
Polyps 10–19 mm, *n* (%)	21 (43.8%)
Polyps ≥ 20 mm, *n* (%)	27 (56.2%)
Location, *n* (%)	
Rectum	5 (10.4%)
Sigmoid colon	3 (6.3%)
Descending colon	4 (8.3%)
Transverse colon	13 (27.1%)
Ascending colon	18 (37.5%)
Cecum	4 (8.3%)
Ileocecal valve	1 (2.1%)
Proximal colon (cecum to transverse)	36 (75.0%)
Distal colon (descending to rectum)	12 (25.0%)
Paris classification, *n* (%)	
0-IIa	33 (68.8%)
0-Is	11 (22.9%)
Is-IIa	4 (8.3%)
JNET classification, *n* (%)	
Type 1	9 (18.8%)
Type 2A	12 (25.0%)
Type 2B	27 (56.4%)
** Resection technique, *n*** ( **%** )	
En bloc cold snare	3 (6.3%)
En bloc hot snare	6 (12.5%)
Piecemeal cold snare	20 (41.7%)
Piecemeal hot snare	15 (31.3%)
Mixed cold/hot snare (piecemeal)	4 (8.3%)
** Histology, *n*** ( **%** )	
Adenoma	37 (77.1%)
Tubular adenoma w/LGD	15
Tubular adenoma w/HGD	6
Tubulovillous adenoma w/LGD	8
Tubulovillous adenoma w/HGD	8
Serrated polyps	9 (18.8%)
SSL without dysplasia	5
SSL with dysplasia	3
Hyperplastic	1
Covert carcinoma (T1)	2 (4.2%)

Data are presented per polyp unless otherwise specified. Values are *n* (%) unless stated otherwise. Histologic subcategories are shown for descriptive completeness and were not analysed separately. Resection technique refers to the index resection method.

LGD = low-grade dysplasia; HGD = high-grade dysplasia; SSL = sessile serrated lesion.

### Initial Resection Techniques

Polyps were resected by cold snare en bloc ( *n* = 3), hot snare en bloc ( *n* = 6), cold snare piecemeal ( *n* = 20), hot snare piecemeal ( *n* = 15) and mixed cold/hot piecemeal ( *n* = 4). Submucosal injection was used in 45/48 cases (93.8%).

### Residual Neoplasia after Standard Resection and CARMA

**Fig. 3:**
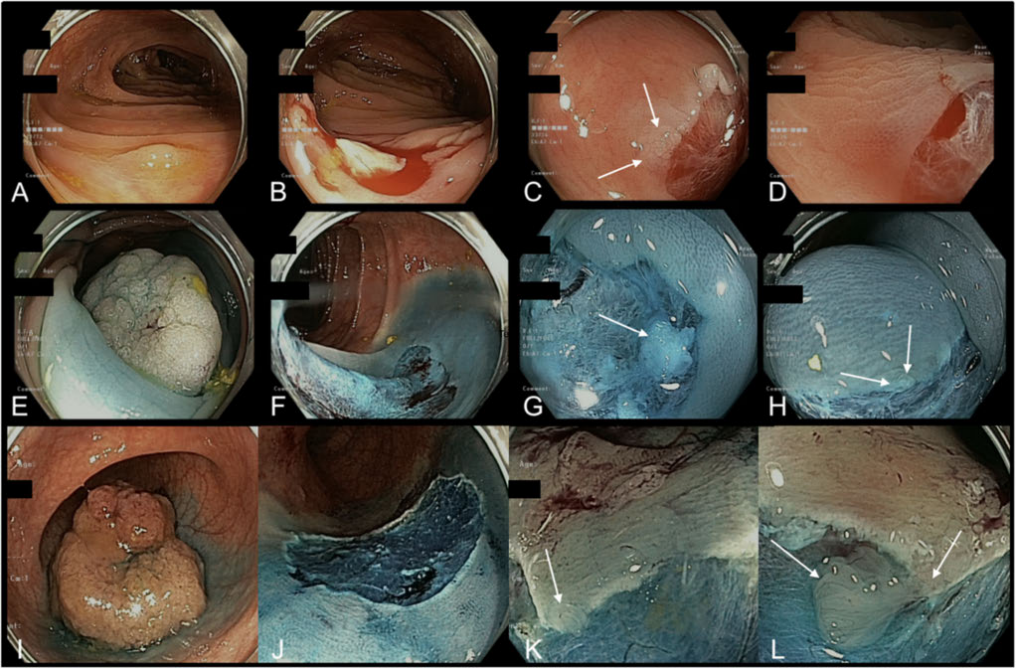
Representative examples of residual neoplasia identified during structured cap-assisted near-field inspection following apparently complete colorectal polyp resection. ( **A** – **D** ) 10-mm sessile serrated lesion (Paris 0-IIa) resected en bloc using cold snare. ( **A** ) Pre-resection appearance. ( **B** ) Post-resection defect under high-definition white light. ( **C** ) Structured cap-assisted near-field inspection demonstrating a focal margin irregularity consistent with residual tissue (arrows). ( **D** ) Water-filled near-field view after targeted cold-snare resection showing a smooth and regular circumferential pit pattern consistent with margin clearance. ( **E** – **H** ) 35-mm granular laterally spreading tumour (Paris 0-IIa), tubulovillous adenoma with low-grade dysplasia, resected piecemeal using cold snare following submucosal injection. ( **E** ) Pre-resection appearance. ( **F** ) Post-piecemeal resection defect. ( **G** ) Cap-assisted inspection demonstrating a discrete base abnormality (arrow). ( **H** ) High-definition near-field view demonstrating subtle margin irregularity (arrow). ( **I** – **L** ) 40-mm tubulovillous adenoma (Paris 0-Is) resected piecemeal using hot snare EMR. ( **I** ) Pre-resection view. ( **J** ) Post-resection defect following submucosal injection and hot snare resection. ( **K** , **L** ) Structured near-field inspection identifying subtle margin irregularities including a villous-appearing residual focus (arrows).

**Fig. 4:**
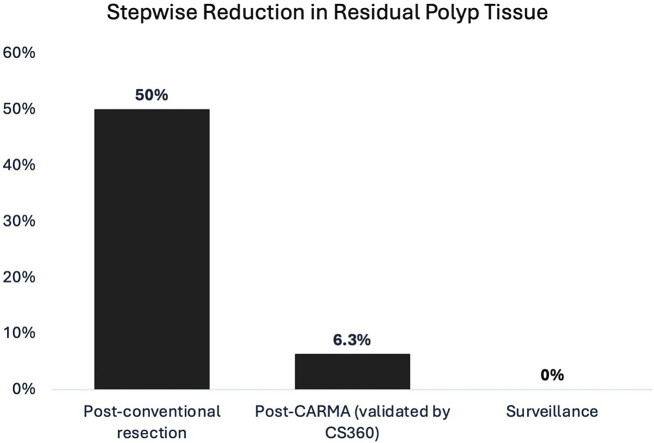
Stepwise reduction in residual disease with CARMA and CS360. Residual polyp tissue was present in 24/48 lesions (50.0%) immediately after standard resection, fell to 3/48 lesions (6.3%) after CARMA confirmed by CS360 and no recurrence was detected at surveillance (0/38 lesions, 0%). The paired reduction from pre- to post-CARMA was statistically significant (absolute risk reduction 43.8%, 95% CI 28.1–57.3; McNemar *p* = 9.5 × 10 ^−7^ ).

Immediately after conventional polyp resection, CARMA identified histology-confirmed residual neoplasia in 24 of 48 polyps (50.0%, 95% CI 35.9–64.1%) ( Fig. 3 ). Targeted cold-snare resection resulted in histology-negative margins in 21 of these 24 lesions. Following CARMA, residual microscopic neoplasia, as detected by CS360, persisted in only 3 of the 48 polyps (6.3%, 95% CI 2.1–16.8%), corresponding to an absolute risk reduction of 43.8% (95% CI 28.1–57.3%; McNemar *p* = 9.5 × 10 ^−7^ ) ( Fig. 4 ).

Applying ESGE guideline criteria (margin thermal ablation recommended for adenomas or sessile serrated lesions with dysplasia ≥20 mm), 24 of 46 non-cancer lesions (52.2%) would have met recommendations for margin ablation (excluding the two lesions with covert cancer). Among the remaining 22 lesions not meeting these criteria, residual neoplasia was identified in 12 (54.5%).

CARMA identified 37 abnormal margin targets (12 high-confidence, 25 low-confidence). High-confidence targets were positive in all cases (PPV 100%, 95% CI 75.8–100%), whereas low-confidence targets were positive in 12 of 25 cases (PPV 48.0%, 95% CI 30.0–66.5%). In the 11 polyps in which CARMA identified no abnormal margin targets, CS360 confirmed complete margin clearance (NPV 100%, 95% CI 71.7–100%).

### CS360 Validation

**Fig. 5:**
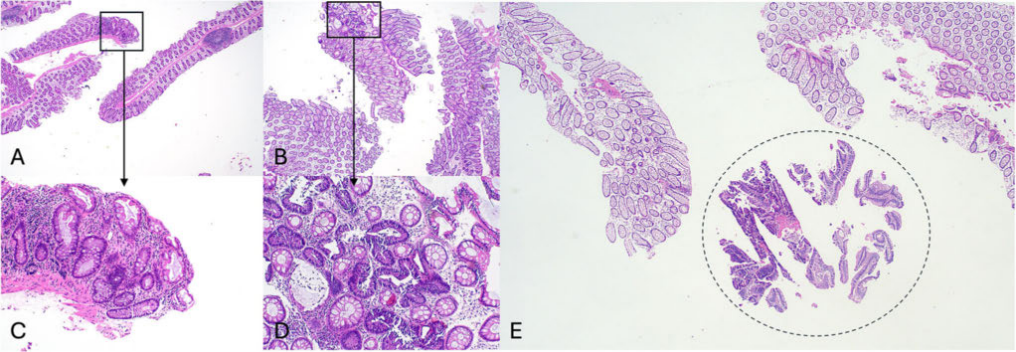
Histologically confirmed residual tissue identified following CARMA-guided margin resection (CS360). ( **A** , **C** ) Box outlines a minute focus of residual sessile serrated lesion (SSL) without dysplasia. The index lesion was a 20 mm SSL with associated adenomatous dysplasia resected by cold-snare piecemeal resection. ( **A** ) Low-power view (4×, H&E); ( **C** ) high-power view (20×, H&E) confirming involvement of four crypts (~0.5 mm). ( **B** , **D** ) Box outlines a small focus of residual tubular adenoma with low-grade dysplasia (TA + LGD). The index lesion was a 16-mm TA + LGD resected by cold-snare piecemeal. ( **B** ) Low-power view (4×, H&E); ( **D** ) high-power view (20×, H&E) demonstrating involvement of approximately 8 crypts (~1 mm). ( **E** ) Circle outlines a single fragmented tubulovillous adenoma (TVA) + LGD tissue fragment without attached normal mucosa likely representing contamination. Index lesion was a 35-mm TVA + LGD removed by hot-snare piecemeal resection. The fragment consisting of diffuse dysplasia only contrasts with the focal crypt-based involvement seen in true residual disease ( **A** – **D** ).

CS360 detected residual microscopic neoplasia after CARMA in 3 of 48 polyps, as described above. One instance was considered possibly attributable to specimen contamination but was conservatively classified as positive; the remaining two consisted of minute, in one case, single-slide foci ( Fig. 5 ).

### Subgroup and Exploratory Inter-operator Analyses

Residual neoplasia was seen to decrease after CARMA across both size strata, all index resection-techniques and both non-cancer histology groups ( Table 2 ). Given small subgroup numbers, post-CARMA percentages should be interpreted cautiously. Post-CARMA residual neoplasia represents immediate microscopic disease detected by CS360 histologic validation and should not be equated with surveillance recurrence.

**Table 2:** Pre- and post-CARMA residual neoplasia rates and absolute reductions across clinically relevant subgroups.

Subgroup	Polyps, *n*	Pre-CARMA residual	Post-CARMA residual	Absolute reduction
Overall cohort	48	24/48 (50.0%)	3/48 (6.3%)	43.8 pp
Size 10–19 mm	21	7/21 (33.3%)	1/21 (4.8%)	28.6 pp
Size ≥20 mm	27	17/27 (63.0%)	2/27 (7.4%)	55.6 pp
Cold snare en bloc	3	2/3 (66.7%)	0/3 (0%)	66.7 pp
Hot snare en bloc	6	2/6 (33.3%)	0/6 (0%)	33.3 pp
Cold snare piecemeal	20	7/20 (35.0%)	2/20 (10.0%)	25.0 pp
Hot snare piecemeal	15	11/15 (73.3%)	1/15 (6.7%)	66.7 pp
Mixed cold/hot piecemeal	4	2/4 (50.0%)	0/4 (0%)	50.0 pp
Adenoma	37	17/37 (45.9%)	2/37 (5.4%)	40.5 pp
Serrated polyp	9	5/9 (55.6%)	1/9 (11.1%)	44.4 pp

Data are presented per polyp. Absolute reduction represents the arithmetic difference between pre-CARMA and post-CARMA residual neoplasia rates, expressed in percentage points. Subgroup analyses were descriptive and not powered for formal between-group comparison. Resection technique was selected according to lesion characteristics and operator judgement. Histology subgroups exclude the two lesions with covert carcinoma; both were pre-CARMA positive and post-CARMA negative. Post-CARMA residual neoplasia represent immediate microscopic disease detected by CS360 histologic validation and should not be equated with surveillance recurrence. pp = percentage points.

**Fig. 6:**
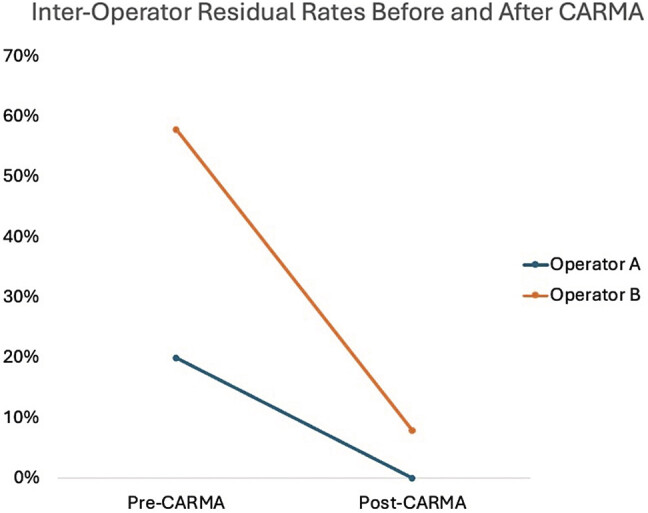
Inter-operator residual rates before and after CARMA. Residual rates differed markedly at baseline (20.0% vs 57.9%, *p* = 0.041), but converged after CARMA (0% vs 7.9%, *p* = 1.0). Lines connect paired before–after measurements for each operator. *CARMA reduced residual tissue for both operators (McNemar p < 0.001).* This analysis was exploratory because only two operators were included and case distribution was unequal.

Pre-CARMA residual rates differed between the two experienced operators, who resected differing numbers of polyps (2/10 {20.0%} vs 22/38 {57.9%}; Fisher’s exact *p* = 0.041). Following CARMA application, residual neoplasia was 0/10 (0%) and 3/38 (7.9%), respectively (Fisher’s exact *p* = 1.0) ( Fig. 6 ).

### Follow-up and Recurrence

Of 44 polyps eligible for surveillance, 38 (86.4%) in 35 patients underwent follow-up colonoscopy at a median of 6 months (range 4–25 months); the denominator structure and reasons for non-eligibility or non-completion are detailed in Fig. 2 . No local endoscopic or histologic recurrence was observed at CARMA scars (0/35 patients; 0/38 polyps; 0%, 95% CI 0–9.3%). Two scars with suspicious endoscopic appearances were resected; both demonstrated only scar fibrosis on histology.

### Adverse Events

One intraprocedural adverse event occurred: a clinically significant bleed during hot-snare resection, which was promptly controlled with snare-tip soft coagulation followed by placement of three haemostatic clips. No intraprocedural adverse events were attributable to CARMA or CS360, and no perforations or delayed bleeding events were observed.

Two patients developed mild-moderate post-procedural abdominal pain (one after cold-snare piecemeal resection and one after hot-snare piecemeal resection). Both were admitted for overnight observation and treated with routine analgesia. Symptoms resolved spontaneously, investigations remained normal and both patients were discharged the following day without further intervention. No patient required re-intervention or unplanned readmission.

## Discussion

### Principal Findings

In this prospective multicentre proof-of-concept study of 48 non-pedunculated colorectal polyps ≥10 mm, histology-confirmed residual neoplasia was present in 50% of lesions immediately after conventional cold- or hot-snare resection deemed complete by the operator. Structured cap-assisted near-field inspection with targeted cold-snare resection (CARMA) reduced residual neoplasia detected by subsequent CS360 validation to 6.3%. Following completion of the full study protocol, no endoscopic or histologic recurrence was observed at surveillance colonoscopy (median 6 months) in 38 (86.4%) evaluable polyps (0%, 95% CI 0–9.3%). In an exploratory analysis, marked inter-operator differences in residual rates before CARMA (20.0% vs 57.9%) appeared to narrow substantially after the protocol was applied (0% vs 7.9%; *p* = 1.0). These findings support incomplete margin clearance as an important modifiable contributor to residual neoplasia and suggest that a structured, auditable, non-thermal margin protocol can reduce residual neoplasia across the resection approaches represented in this cohort.

These observations are directionally consistent with prior evidence that margin-directed treatment significantly reduces post-polypectomy recurrence and extend this concept by providing a structured, histology-linked approach to immediate margin assessment and clearance. 12 14

Notably, residual neoplasia was also frequently observed in lesions that would not meet current guideline criteria for routine thermal margin ablation, underscoring that incomplete margin clearance extends beyond traditionally high-risk subgroups. 19

### Mechanisms of Residual Disease and Limitations of Conventional Inspection

The high prevalence of residual neoplasia after apparently complete conventional resection reflects three structural deficiencies: (i) operator-dependent margin assessment, (ii) absence of a validated intra-procedural endpoint and (iii) systematic under-sampling when only a few margin biopsies are taken. 5 By stabilising the mucosal view with a distal cap and mandating near-field inspection of the entire circumference, CARMA identified both unequivocally abnormal high-confidence targets and low-confidence margin areas in which a normal pit-pattern endpoint could not be confirmed. High-confidence targets were uniformly positive, whereas nearly half of low-confidence targets also contained histologically confirmed residual neoplasia. Low-confidence resection therefore represents a targeted sensitivity–specificity trade-off: some histologically negative tissue may be removed to achieve a verifiable normal pit-pattern endpoint, while reducing the risk of leaving residual neoplasia untreated. Unlike circumferential margin treatment, this additional resection was limited to abnormal or non-verifiable margin areas. CS360-positive findings after CARMA were rare (3/48 polyps) and were limited to minute microscopic foci, including one isolated fragment interpreted as a likely contaminant. These findings suggest that much residual neoplasia may be endoscopically appreciable when assessed using structured, stabilised near-field inspection.

Few prior margin-treatment strategies have combined a structured, circumferential approach to margin management with protocolisation and the capacity for histologic confirmation of clearance. 19 20

### Complementary Roles of CARMA and CS360

This study introduces two complementary, non-thermal strategies that provide structured approaches to margin inspection and/ or clearance with histologic verification.

CARMA is an image-guided protocol that combines mechanical stabilisation (distal cap), near-field optics and a mandatory response to any abnormal pit pattern. Its endpoint – a circumferentially normal pit-pattern margin – is visually verifiable, teachable and directly linked to histologic outcome. CARMA can be layered onto any existing resection technique (cold snare, hot snare, en bloc or piecemeal) without additional equipment beyond a standard transparent cap. Existing adjuncts to margin management have largely focused on post-resection thermal ablation **,** which can markedly reduce recurrence but does not provide a histologic quality metric, remains operator-dependent and is currently recommended only for selected colorectal polyps. 6 19

CS360 is a mechanical, protocolised 2–3 mm circumferential cold-snare excision of the post-resection margin. In the present study, CS360 served primarily as a circumferential histologic reference standard after CARMA; its role as a stand-alone margin treatment requires dedicated evaluation. In contrast to approaches that aim for margin security through wide-margin excision, pre-resection circumferential incision *or* thermal destruction **,** CS360 yields histology and standardises circumferential treatment independent of optical recognition. 31 32

Low residual rates were observed after CARMA, while CS360 may have roles where maximal histologic assurance or training validation is desired.

### Exploratory Analysis of Inter-operator Variability

Marked inter-operator variability in recurrence and incomplete resection is one of the most consistent findings in large EMR cohorts, with differences exceeding threefold even among experts. 6 7 8 In the current study, two experienced endoscopists using identical equipment showed a near-threefold disparity in residual neoplasia after conventional resection, comparable to the inter-operator variability reported in the CARE study. 5 Following application of the CARMA protocol, this gap appeared to narrow substantially, supporting the hypothesis that a standardised, margin-focused endpoint may reduce operator-dependent differences in resection completeness. However, this observation was based on only two operators and unequal case distribution and should therefore be interpreted as exploratory.

Other margin-directed strategies, including thermal ablation and wide-margin excision, have also demonstrated reductions in recurrence and variability in expert settings; however, these approaches are either modality-restricted or lack a universally applicable, verifiable endpoint for margin completeness.

These findings align with prior calls for validated quality metrics for completeness of resection. 5 33 A structured, auditable margin endpoint may support future quality-assurance frameworks but requires validation in larger operator cohorts.

### Clinical Implications

This proof-of-concept study introduces CARMA as a structured margin-assessment and targeted-clearance protocol designed to improve post-resection margin management. CARMA is not proposed as a replacement for guideline-recommended thermal margin ablation, and procedure time, learning curve, cost, workflow burden, safety and reproducibility outside expert cap-assisted operators were not assessed. The validation workflow, including CS360 and separate specimen handling, would not form part of routine use outside audit, training or research. CS360 served as circumferential histologic validation in this study and may warrant dedicated evaluation as a protocolised cold-snare margin-excision strategy in training, quality-improvement programmes or selected high-risk lesions. Practical feasibility, scalability and effects on clinical recurrence therefore require evaluation in larger pragmatic studies. 13 33 34

### Strengths and Limitations

Strengths include the prospective multicentre design, separate specimen retrieval with cut-to-extinction histology, predefined protocol and CS360 as an unbiased, circumferential histologic reference standard. Detailed mapping of targeted resections and systematic inter-operator comparison provide unique mechanistic insight into the origins of residual disease.

Limitations include modest sample size, clinically selected resection technique and performance by two highly experienced cap-assisted endoscopists. Subgroup and inter-operator findings should be interpreted descriptively rather than as comparative efficacy analyses, and generalisability to less specialised settings remains uncertain. The deliberately heterogeneous lesion and resection method mix reflects the intended broad applicability of CARMA across routine polypectomy practice, but limits formal inference within individual lesion or resection subgroups. Surveillance was relatively short (median 6 months); however, >90% of post-EMR recurrences are reported within the first year, and the absence of early recurrence should be interpreted in this context. 35 The clinical significance of the three microscopic foci detected only by CS360 remains uncertain and was classified conservatively.

### Future Directions

Larger pragmatic studies are required to assess reproducibility across broader operator groups, lesion characteristics and practice settings; define learning curves and protocol adherence; and determine whether immediate residual-neoplasia reduction translates into long-term recurrence reduction.

## Conclusion

Structured cap-assisted margin assessment with targeted cold-snare resection substantially reduced immediate histology-confirmed residual neoplasia after colorectal polypectomy in this proof-of-concept study. Much of this residual neoplasia was endoscopically visible under structured near-field inspection and amenable to targeted resection, challenging the assumption that post-resection residual disease is necessarily microscopic or endoscopically inapparent. CS360 provided circumferential histologic validation after CARMA and identified only uncommon minute residual microscopic foci; it may also represent a protocolised cold-snare margin-excision strategy that warrants dedicated evaluation. Together, CARMA and CS360 provide complementary non-thermal approaches to verifiable margin-focused assessment and clearance. Larger real-world studies are required to assess durability, implementation and potential effects on inter-operator variability.

## Data Availability

Individual participant data will not be shared. Aggregate data supporting the findings of this study are available from the corresponding author upon reasonable request and subject to institutional ethics and data governance approval.
